# From hepatitis misdiagnosis to zoonotic false alarms: a metagenomic blacklist framework for the parvo-like hybrid viral group

**DOI:** 10.1128/spectrum.00157-26

**Published:** 2026-04-09

**Authors:** Peng Zhao, Hong Liu, Jie Dong, Haoxiang Su, Qi Jin, Fan Yang

**Affiliations:** 1 NHC Key Laboratory of Systems Biology of Pathogens, National Institute of Pathogen Biology, Chinese Academy of Medical Sciences & Peking Union Medical College 12501, Beijing, China; 2 Shandong Provincial Research Center for Bioinformatic Engineering and Technique, School of Life Sciences and Medicine, Shandong University of Technology 91620, Zibo, China; 3 Key Laboratory of Respiratory Disease Pathogenomics, Chinese Academy of Medical Sciences, Beijing, China; 4 State Key Laboratory of Respiratory Health and Multimorbidity, Beijing, China; The Ohio State University College of Veterinary Medicine, Columbus, Ohio, USA

**Keywords:** metagenomic sequencing, parvo-like hybrid viruses (PHVs), reagent contamination, false-positive signals, silica membrane spin columns, pathogen surveillance bias, PVDDC

## LETTER

Metagenomic sequencing has become a cornerstone of pathogen discovery and emerging infectious disease surveillance, enabling unbiased detection of known and unknown viruses directly from clinical, animal, and environmental samples (1–5). However, the same sensitivity that underpins these approaches also increases susceptibility to laboratory-derived nucleic acid contaminants, which may be misinterpreted as evidence of novel viral circulation or cross-species transmission (6, 7). Such artifacts pose a particular challenge for early-warning systems, where spurious signals can complicate biological interpretation and downstream risk assessment.

Parvo-like hybrid viral (PHV) sequences exemplify this problem. One representative strain, NIH-CQV, was initially reported as a candidate etiological agent of seronegative hepatitis (8) but was subsequently shown to originate from nucleic acid contaminants embedded in silica-based extraction reagents (7, 9, 10). Despite this clarification, related PHV sequences continue to be detected in metagenomic data sets from diverse wildlife and environmental sources and are frequently annotated as putative infectious or zoonotic viruses, sustaining uncertainty regarding their biological relevance (6, 11).

**Fig 1:**
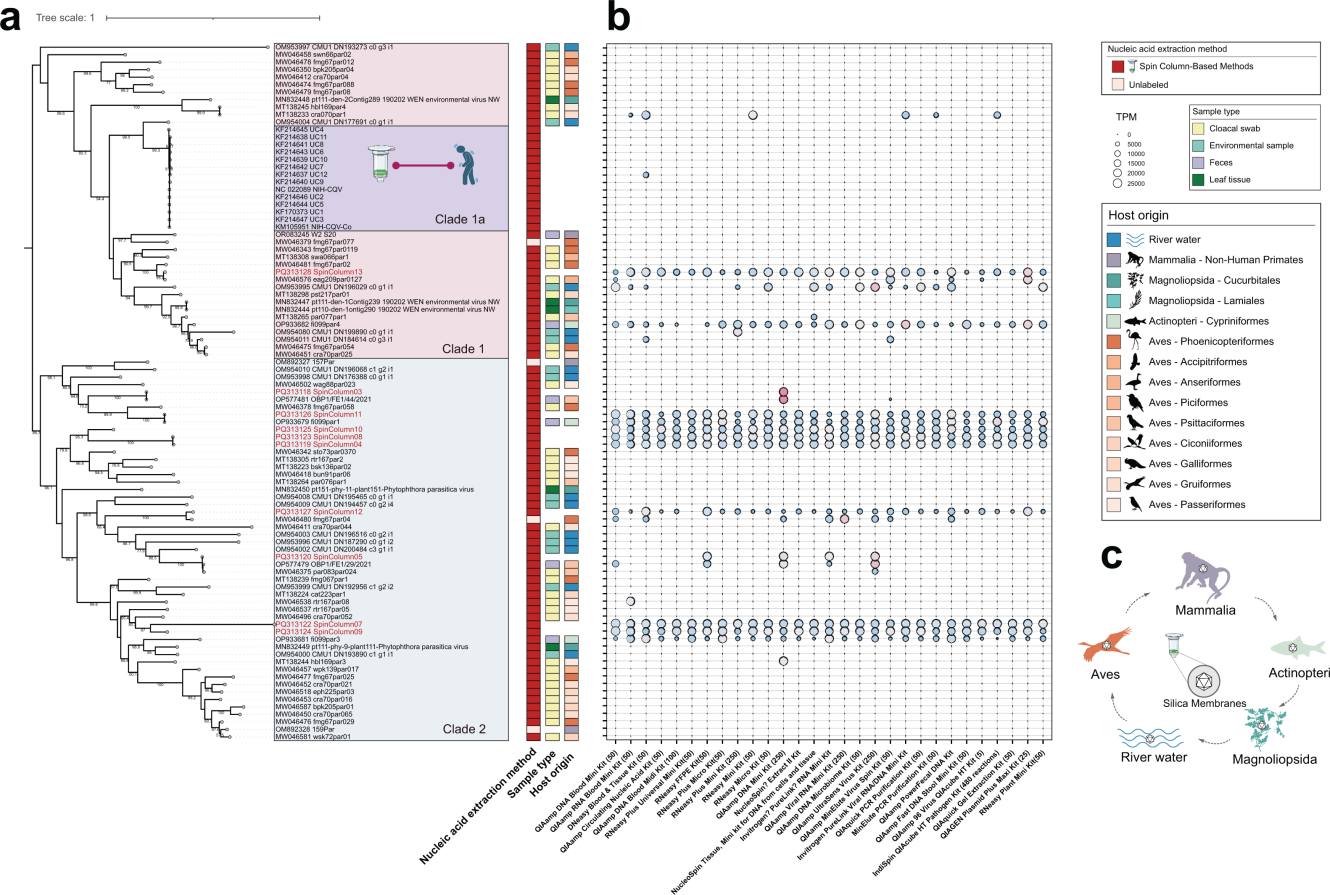
Evidence that parvo-like hybrid viral sequences are reagent-derived contaminants. (**a**) Maximum-likelihood phylogeny of parvo-like hybrid viral (PHV) sequences inferred from NS amino acid alignments using the LG+R5 substitution model with midpoint rooting. PHV sequences newly identified in this study are highlighted in red. Colored strips indicate the nucleic acid extraction method, biological sample type, and reported host or source associated with each sequence. (**b**) Bubble plot showing the abundance of PHVs detected in metagenomic data sets generated from silica membranes of 29 commercial spin column-based nucleic acid extraction kits. (**c**) Conceptual illustration highlighting the ecological implausibility of interpreting PHV sequences as evidence of cross-species viral infections across highly divergent host taxa.

Here, we provide systematic evidence that PHV sequences detected by metagenomics represent reagent-derived artifacts rather than genuine infectious viruses. Using the Panoramic Virus Discovery Data Chain (PVDDC) developed by us (6), we curated 93 global PHV genomes with detailed metadata, including 10 newly assembled sequences recovered directly from silica membranes of 29 commercial nucleic acid extraction kits representing diverse manufacturers and intended applications (Table S1). Phylogenetic analysis resolved two major clades spanning substantial sequence divergence (Fig. 1a); however, neither clade exhibited ecological structure consistent with host taxonomy, sampling context, or geographic origin. Instead, sequences recovered from extraction reagents were interspersed throughout both clades alongside PHVs previously attributed to birds, fish, plants, and other hosts (Fig. 1b).

Multiple PHVs reported as wildlife-associated viruses showed implausibly high nucleotide identity (>90%–95%) to sequences obtained directly from extraction reagents. Such extreme sequence conservation across highly divergent host taxa is incompatible with established principles of viral evolution and host adaptation and cannot be readily explained by repeated natural spillover events (Fig. 1c). Similar contamination-driven host misassignments have been documented for other parvovirus-related sequences (6), suggesting that PHVs represent a prominent manifestation of a broader technical artifact affecting metagenomic virome studies.

Consistent with this interpretation, metagenomic profiling of silica membranes from 29 commercial spin column-based nucleic acid extraction kits revealed widespread and recurrent detection of PHV sequences independent of biological sample input (Fig. 1b). Several PHVs were detected in the majority of kits tested, indicating propagation at industrial scale through reagent supply chains. Detection of PHVs in sequencing blanks further confirms that laboratory consumables alone are sufficient to generate these signals (7, 9, 10).

Taken together, the absence of ecological coherence, the implausibly high sequence identity across highly divergent hosts, and the pervasive association with nucleic acid extraction reagents demonstrate that the vast majority of PHV sequences reported to date represent laboratory-derived artifacts rather than bona fide infectious viral entities. While we cannot completely exclude the possibility that rare PHV-related sequences may have a biological origin, our data indicate that the signals currently dominating public databases and metagenomic studies arise from reagent contamination. Continued interpretation of these sequences as infectious or zoonotic viruses risks propagating non-biological records in virology databases and confounding metagenomic-based pathogen discovery and emerging infection surveillance. We therefore recommend that PHV sequences be interpreted with caution and flagged as probable reagent-derived artifacts until definitive biological hosts are identified.

## Data Availability

The metagenomic sequencing data from the silica membrane virome have been deposited in the NCBI Sequence Read Archive under BioProject accession number PRJNA1158863. All sequences generated in this study have been submitted to GenBank under accession numbers PQ313118–PQ313128. Details of library construction are described in our previous study (6), and the phylogenetic analysis and metagenomic data processing are provided in the Supplemental material.

## SUPPLEMENTAL MATERIAL

Supplemental material spectrum.00157-26-s0001.docx Supplemental methods related to data analysis.

Table S1 spectrum.00157-26-s0002.xlsx Overview of the 29 commercial nucleic acid extraction kits analyzed in this study.

ASM does not own the copyrights to Supplemental Material that may be linked to, or accessed through, an article. The authors have granted ASM a non-exclusive, world-wide license to publish the Supplemental Material files. Please contact the corresponding author directly for reuse.
