# Supplementary material for: From hepatitis misdiagnosis to zoonotic false alarms: a metagenomic blacklist framework for the parvo-like hybrid viral group
Source: Microbiol Spectr. 2026 Apr 9;14(5):e00157-26. doi: 10.1128/spectrum.00157-26 (PMC13141889; doi:10.1128/spectrum.00157-26)
Supplement: Supplemental material — Supplemental methods related to data analysis. [file spectrum.00157-26-s0001.docx]

Extended Methods

Metagenomic Sequencing and Data Analysis

The preparation and pretreatment procedures for silica membrane samples used in nucleic acid extraction kits followed protocols established in our previous study(1).

Raw reads generated from high-throughput sequencing were first subjected to quality filtering using Trimmomatic (v0.39) to eliminate adaptor sequences and low-quality base calls(2). Cleaned reads were subsequently assembled into contigs using both SPAdes (v3.15.4)(3) and MEGAHIT (v1.2.9)(4), and contigs shorter than 480 bp were discarded from downstream analysis. To identify viral sequences, contigs were searched against the NCBI non-redundant (nr) protein database using DIAMOND Blastx (v0.9.31.132), with an e-value cutoff of 1×10⁻⁵ and a minimum subject coverage of 60%(5). Viral hits were inspected and extracted using MEGAN (v6.25.10)(6). Additional refinement was performed through online BLASTn and BLASTx searches to retrieve the most closely related parvo-like hybrid virus sequences(7). To minimize redundancy, retrieved viral contigs were clustered using CD-HIT (v4.8.1) at a 90% sequence identity threshold(8). For abundance quantification, short reads were mapped to the 93 PHV reference sequences using Bowtie2 (v2.5.1) in the “--very-sensitive” mode(9), and alignments yielding fewer than three reads per library were excluded to reduce potential cross-library contamination.

The metagenomic sequencing data from the silica membrane virome have been deposited in the NCBI Sequence Read Archive under BioProject accession number PRJNA1158863. All sequences generated in this study have been submitted to GenBank under accession numbers PQ313118–PQ313128.

Phylogenetic Analysis and Visualization

Multiple sequence alignment was performed using the auto mode in MAFFT (v7.450)(10). To improve alignment quality, ambiguous regions were removed using TrimAl (v1.5)(11), and the resulting alignments were inspected and curated manually in Geneious Prime (v2023.0.1). Maximum likelihood (ML) phylogenetic trees were constructed with IQ-TREE (v2.3.6), where the optimal substitution model was selected using the built-in model finder option (-m MFP). Branch support was estimated using 1,000 replicates of the SH-like approximate likelihood ratio test (SH-aLRT)(12). Final tree topologies were visualized using Interactive Tree of Life (iTOL) (v6)(13).

**SI References**

1. Zhao P, Dong J, Liu H, Team DRaC, Xue Y, Zhang T, Sui H, Hu Y, Du J, Su H, Jin Q, Yang F. 2025. Unveiling Origin Pitfalls in Metagenomic Surveillance for Emerging Infectious Diseases: Parvoviruses as a Model. iMetaMed 1:e70004.

2. Bolger AM, Lohse M, Usadel B. 2014. Trimmomatic: a flexible trimmer for Illumina sequence data. Bioinformatics 30:2114-20.

3. Bankevich A, Nurk S, Antipov D, Gurevich AA, Dvorkin M, Kulikov AS, Lesin VM, Nikolenko SI, Pham S, Prjibelski AD, Pyshkin AV, Sirotkin AV, Vyahhi N, Tesler G, Alekseyev MA, Pevzner PA. 2012. SPAdes: a new genome assembly algorithm and its applications to single-cell sequencing. J Comput Biol 19:455-77.

4. Li D, Luo R, Liu CM, Leung CM, Ting HF, Sadakane K, Yamashita H, Lam TW. 2016. MEGAHIT v1.0: A fast and scalable metagenome assembler driven by advanced methodologies and community practices. Methods 102:3-11.

5. Buchfink B, Xie C, Huson DH. 2015. Fast and sensitive protein alignment using DIAMOND. Nat Methods 12:59-60.

6. Huson DH, Beier S, Flade I, Gorska A, El-Hadidi M, Mitra S, Ruscheweyh HJ, Tappu R. 2016. MEGAN Community Edition - Interactive Exploration and Analysis of Large-Scale Microbiome Sequencing Data. PLoS Comput Biol 12:e1004957.

7. Altschul SF, Madden TL, Schaffer AA, Zhang J, Zhang Z, Miller W, Lipman DJ. 1997. Gapped BLAST and PSI-BLAST: a new generation of protein database search programs. Nucleic Acids Res 25:3389-402.

8. Fu L, Niu B, Zhu Z, Wu S, Li W. 2012. CD-HIT: accelerated for clustering the next-generation sequencing data. Bioinformatics 28:3150-2.

9. Langmead B, Salzberg SL. 2012. Fast gapped-read alignment with Bowtie 2. Nat Methods 9:357-9.

10. Rozewicki J, Li S, Amada KM, Standley DM, Katoh K. 2019. MAFFT-DASH: integrated protein sequence and structural alignment. Nucleic Acids Res 47:W5-W10.

11. Capella-Gutierrez S, Silla-Martinez JM, Gabaldon T. 2009. trimAl: a tool for automated alignment trimming in large-scale phylogenetic analyses. Bioinformatics 25:1972-3.

12. Minh BQ, Schmidt HA, Chernomor O, Schrempf D, Woodhams MD, von Haeseler A, Lanfear R. 2020. IQ-TREE 2: New Models and Efficient Methods for Phylogenetic Inference in the Genomic Era. Mol Biol Evol 37:1530-1534.

13. Letunic I, Bork P. 2021. Interactive Tree Of Life (iTOL) v5: an online tool for phylogenetic tree display and annotation. Nucleic Acids Res 49:W293-W296.
